# Flexibility and inflexibility in an Iranian sample: psychometric properties of MPFI based on the Hexaflex model

**DOI:** 10.1186/s40359-024-01531-0

**Published:** 2024-01-18

**Authors:** Mahboobeh Moosivand, Mohammad Javad Bagian Kulemarzi, Maryam Sayad Shirazi, Zeinab Zaremohzzabieh

**Affiliations:** 1 Department of Social Science and Development Studies, Women Research Center, Alzahra University, Tehran, Iran; 2 Razi University, Kermanshah, Iran; 3 Department of Psychology, Women Research Center, Alzahra University, Tehran, Iran; 4 Institute for Social Science Studies, Universiti Putra Malaysia, Serdang, Selangor Malaysia

**Keywords:** Acceptance and commitment therapy (ACT), Flexibility and inflexibility, Hexaflex model

## Abstract

**Objectives:**

Acceptance and Commitment Therapy (ACT) emphasizes the importance of psychological flexibility in promoting emotional, psychological, and social well-being, while also acknowledging rigidity as a precursor to psychological disorders. Analyzing the psychometric qualities based on the multidimensional Hexaflex model is critical for determining the efficiency of therapeutic interventions. Thus, the purpose of this study is to investigate the psychometric features of the Multidimensional Psychological Flexibility Inventory (MPFI) within the context of the Hexaflex model in a group of Iranian university students.

**Methods:**

Exploratory and confirmatory factor analyses were used in this study to evaluate the psychometric features of the flexibility/inflexibility scale (MPFI) in a sample of Iranian university students.

**Findings:**

In the exploratory factor analysis involving a sample of 300 students, six factors were identified for flexibility and six for inflexibility (56.3% males and 43.7% females). In the confirmatory factor analysis with a sample of 388 participants, the results validated 60 items across a total of six flexibility and inflexibility factors. This outcome can serve as a robust estimate for flexibility, inflexibility, the second-order model, and the final model. Cronbach’s alpha values for various components, including acceptance, present-moment awareness (or contact with the present moment), self as context, cognitive defusing, values, committed action, total flexibility, experiential avoidance, lack of present-moment awareness, self as content, fusion, lack of contact with values, inaction, and total inflexibility, were reported as follows: 0.818, 0.869, 0.862, 0.904, 0.935, 0.935, 0.942, 0.895, 0.839, 0.883, 0.904, 0.912, 0.941, and 0.941, respectively.

**Conclusions:**

The Farsi version of the MPFI for university students has great psychometric qualities, making it a reliable assessment instrument for the ACT.

## Introduction

The Psychological Flexibility (PF) model, a promising transdiagnostic framework for understanding psychopathological processes, holds a pivotal position in mental health research [1]. At its core, PF is defined as the capacity to maintain a full psychological presence, accept all thoughts and emotions, and actively engage in behaviors aligned with one’s values. This conceptualization typically encompasses six fundamental processes, intricately described within the framework of Acceptance and Commitment Therapy (ACT) [2]. In contrast, Psychological Inflexibility (PI) embodies maladaptive behaviors that hinder the enhancement or sustenance of an individual’s well-being and is postulated as a significant source of psychopathology within the ACT framework [2].

Concurrently, the social framework hypothesis introduces an additional dimension to PF, emphasizing its role in enabling individuals to embrace their experiences and align their actions with their core life values through present-moment awareness [3]. Individuals characterized by high PF levels tend to cope more effectively with distressing thoughts, emotions, and challenging life situations, ultimately contributing to an overall improvement in their well-being [4]. Furthermore, PF has been identified as a protective factor that modifies the link between everyday life stress, physical functioning, and mental health. It’s worth noting that ACT, built upon the fundamental psychological constructs of flexibility and inflexibility [5], places PF at the heart of this transformative process [6]. ACT is rooted in the contextual behavior sciences and draws from the conceptual framework provided by Relational Frame Theory (RFT) [3], an influential behavioral theory that analyzes relational framing as the fundamental process underpinning human language and cognition [7]. The Hexaflex model, proposed within ACT, provides a comprehensive understanding of the primary processes that underlie human suffering and psychopathology [4]. Hayes et al. [2] describe PF as a multi-dimensional model of psychopathology, psychological health, and psychological intervention.

Individuals are equipped with PF to experience ideas, feelings, and experiences while actively making decisions and performing actions that are consistent with their values [4]. It is built around six fundamental ACT processes: acceptance, defusion, present moment awareness, self-as-content, committed action, and values. Conversely, PI is composed of six opposing dimensions: lack of present-moment awareness, lack of contact with values, inaction, self-as-content, fusion, and experiential avoidance [8]. PI, a construct closely linked to the development and persistence of various psychological issues, has been shown to correlate with a range of psychological problems, such as somatization, depression, anxiety, and posttraumatic stress symptoms [9]. A study by Levin et al. [10] revealed significant correlations between PI and a variety of psychological and behavioral problems, including depressive symptoms, anxiety, and eating disorders.

While the importance of PF has increased, the measurement of this construct and its numerous dimensions has lagged behind the theory’s practical and clinical implications. Most widely used psychological flexibility measures, such as the Acceptance and Action Questionnaire (AAQ), the Acceptance and Action Questionnaire-II (AAQ-II), and the Avoidance and Fusion Questionnaire for Adolescents (AFQY), treat PF as a single factor, despite the theory suggesting the presence of up to 12 distinct dimensions. Some scales have been developed, incorporating different elements of the Hexaflex model, such as the Mindful Attention and Awareness Scale (MAAS). However, these scales vary in size and complexity and lack a comprehensive scale that directly encapsulates all 12 factors following the Hexaflex model. Evaluating the psychometric properties of such a scale complements the investigation of PF dimensions, thereby assisting in the identification of aspects that are pivotal for well-being and health, that are directly impacted by medical interventions, and that can act as moderators influencing the effectiveness of the treatment process.

Previous measurements of PI have established the foundation for assessing flexibility by amalgamating the model’s 12 dimensions into a single composite measure, emphasizing the common variance shared among the 12 dimensions within the Hexaflex model [11–13]. This approach aligns with the Hexaflex model, which posits intricate interconnections between the various dimensions of flexibility. Nevertheless, these measures are designed to evaluate clinical and research psychologists in general, rather than assessing the specific elements put forth in the Hexaflex model. In recent years, some multidimensional scales have made headway by utilizing specific components of the Hexaflex model to assess its core concepts, thereby contributing significantly to this field (e.g., the Multidimensional Experience Avoidance Questionnaire - MEAQ [14], Attention Questionnaire Five-Facet Scale - FFMQ [15], Self-Compassion Scale - SCS) [16].

**Fig. 1:**
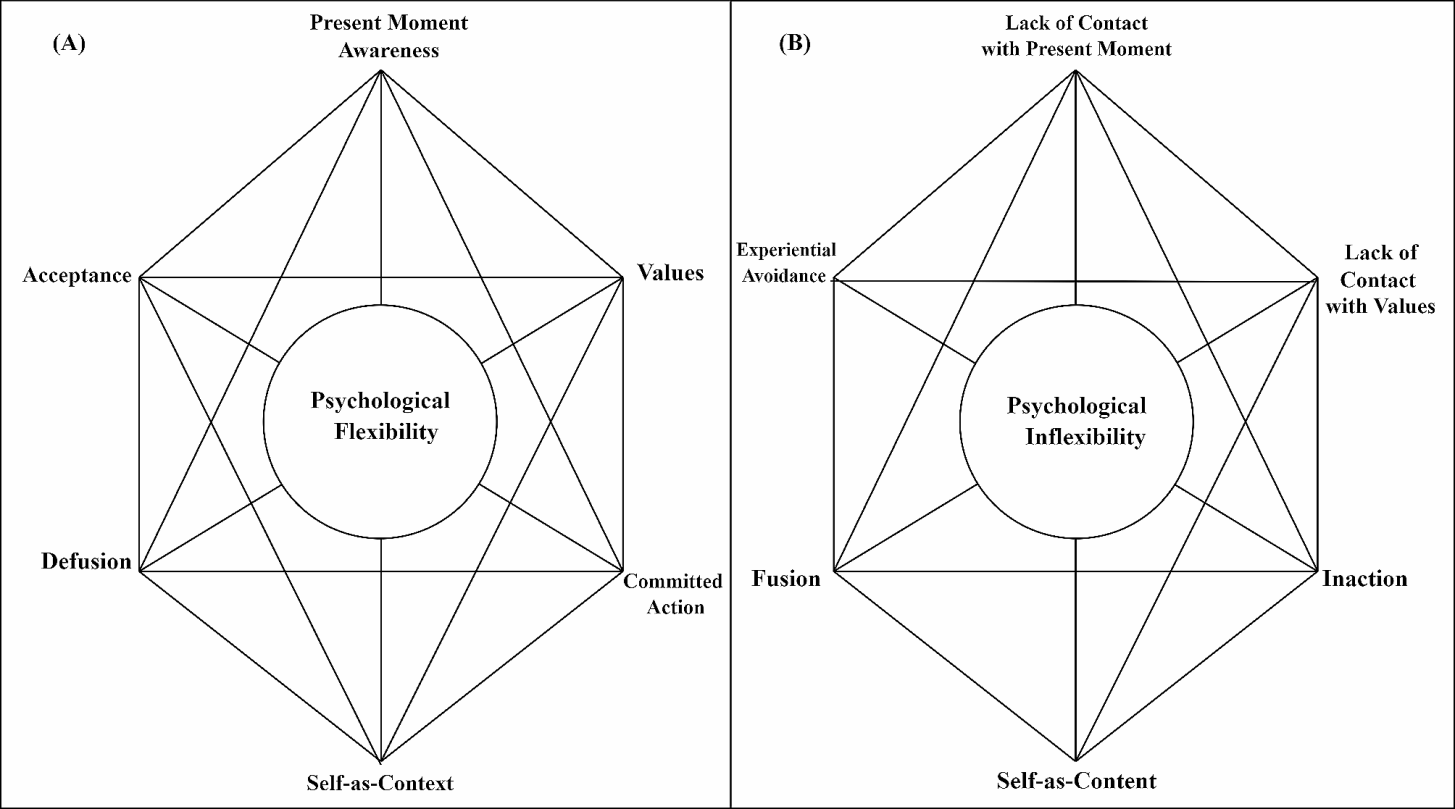
Model of psychological flexibility (Hexaflex) [3]

In contrast to PF, PI signifies the overwhelming influence of psychological reactions and unpredictable conditions over chosen values and actions [12]. Consequently, PF is the response to unwanted personal experiences, encompassing thoughts, feelings, and physical sensations [3]. PF consists of six distinctive components: acceptance (a willingness to fully engage in unfavorable experiences), awareness of the present moment (maintaining an intimate connection with and awareness of one’s experiences), self-as-context (maintaining one’s perspective within the context of one’s experiences), cognitive defusion (the ability to disentangle oneself from unwanted experiences without becoming ensnared in them), committed action (sustaining behaviors that align with vital aspects of life), and values (maintaining a connection with crucial areas of life that provide direction to one’s behaviors) [12]. The model also postulates six specific components of PI: experiential avoidance (attempts to create distance from unwanted experiences), cognitive fusion (becoming entangled in undesirable inner experiences), inaction (inability to behave in harmony with what is crucial in life), transient unconsciousness (disregarding one’s experiences at a given moment), the self as content (passing judgments about experiences that lead to a narrower self-perspective), and lack of contact with values (disconnection from the most meaningful aspects of life) [4]. It’s essential to note that while these dimensions are conceptually separate, the Hexaflex model suggests that the 12 components of flexibility and inflexibility have robust mutual relationships (Fig. 1).

Research indicates that mental inflexibility is linked to higher levels of depression, anxiety, and obsessive-compulsive tendencies [10]. Moroz and Dunkley’s [17] two-year study revealed that experience avoidance increased alongside symptoms of depression and anxiety, providing further support for the idea that avoiding unwelcome thoughts and emotions, or internal experiences, contributes to heightened stress levels.

On a practical level, the Hexaflex model provides researchers with a palette of 12 distinct change mechanisms to investigate the efficacy of ACT. It furnishes a comprehensive theoretical framework to dissect the therapeutic approach and pinpoint the critical components responsible for meaningful transformations. The Hexaflex model provides a collection of 12 unique elements that clients bring to treatment. As a result, it not only contributes to a deep understanding of the distinctions between closely related conditions (e.g., major depression versus major depressive disorder), but it also provides 12 dimensions that can be investigated as potential moderators of treatment responses (e.g., identifying individuals who are most and least likely to benefit from treatment) [18]. While the Hexaflex model is more closely tied to ACT, it might also be utilized to investigate the mechanics of other treatment methods. The Hexaflex model provides a theoretical framework for foundational research on a wide range of individual and interpersonal outcomes, including health and wellness habits. However, to fully exploit the potential benefits of the Hexaflex paradigm, a multidimensional PF scale must be developed.

Rolffs et al. [4] took the lead in developing and validating the Multidimensional Psychological Flexibility Inventory (MPFI), a 60-item scale, via three large-scale investigations involving 3040 people in the United States. This measure was meticulously constructed from a pool of 554 potential items, most of which were sourced from 22 widely recognized measures within the ACT and mindfulness literature. The initial study, which involved 372 participants, conducted both exploratory and confirmatory factor analyses to establish the factor structure of the MPFI measure. In the second study, which had a sample size of 2150 participants, item response theory (IRT) was used to analyze a refined set of 288 items, ultimately selecting the top five most effective indicators for each subcomponent of psychological flexibility and inflexibility. The conclusive exploratory and confirmatory factor analyses revealed a hierarchical factor structure, where global psychological flexibility and inflexibility were identified as second-order factors, while the respective subcomponents were categorized as first-order factors. In the third study, which included 518 participants, strong evidence of convergent validity was found, as the MPFI’s subscales showed robust correlations with established measures of psychological flexibility and inflexibility. When it came to discriminant validity, the MPFI’s subscales displayed comparatively weaker correlations with conceptually different constructs, such as emotional intelligence, neuroticism, curiosity, need satisfaction, and psychological distress. The MPFI’s 12 subscales exhibited exceptional internal consistency, with Cronbach’s alpha values ranging from 0.87 to 0.97 consistently across various demographic subgroups, including different genders, age groups, ethnic backgrounds, and mental health statuses [4].

A subsequent replication study conducted by Landi et al. [19] affirmed the factor structure of the original MPFI through confirmatory factor analysis. The Italian adaptation of the MPFI unveiled a two-tiered factor structure, consisting of six first-level factors associated with flexibility and another six first-level factors related to inflexibility. This analysis provided evidence of strong construct validity. The Italian version of the MPFI showcased impressive internal consistency, along with strong evidence of convergent and concurrent validity. Additionally, it exhibited measurement invariance across gender, age, and mental health status [19]. Tabrizi et al. [7] emphasized that the MPFI comprises 12 subscales, six of which evaluate flexibility and the remaining six assess inflexibility. A confirmatory factor analysis conducted with a community sample of 670 participants supported the validity of a model with two higher-order factors (CFI = 0.933), while a 12-factor model demonstrated the best fit (CFI = 0.955). Each of the 12 subscales demonstrated reliability within an acceptable range (composite reliability = 0.803–0.933), and the factor structure remained stable across various age groups and genders.

Against this backdrop, our study addresses a significant gap in the current literature by evaluating the 60-item MPFI within the Hexaflex model. Through two distinct investigations, we simultaneously assess the validity of flexibility and inflexibility dimensions, incorporating the second version of AAQII and exploring psychological factors such as anxiety, stress, and depression. The emphasis on Iranian university students is justified due to the documented increase in depression, anxiety, and stress within this demographic [20, 21]. Our goal is to provide valuable insights into the intricate stressors faced by Iranian university students, including academic pressures, social interactions, and personal growth. By investigating psychological flexibility through the MPFI, our research aims to offer a nuanced understanding of how students navigate these challenges, aligning with the core components of the Hexaflex Model. This targeted approach yields valuable insights into the psychological processes crucial for academic success, well-being, and personal development during university life. Notably, previous studies validating the MPFI have predominantly focused on clinical and Iranian community samples of adults [22, 23]. The present study seeks to bridge this gap by specifically investigating the psychometric features of the MPFI within the Hexaflex model in the context of Iranian university students, addressing a significant oversight in prior research on this demographic.

## Methods

### Study design and participants

The first survey was conducted between June and August 2020, and the second survey took place in June 2021. The participants were drawn from first-year postgraduate co-curricular programs at public universities in Iran. A cluster sampling design was employed to collect self-reported data from students currently enrolled in courses within the computer, Humanities and Social Sciences, and medical science departments.

In the first study, 300 university students participated, consisting of 169 males (56.3%) and 131 females (43.7%). Their ages ranged from 20 to 45 years, with an average age of 24.67 and a standard deviation of 5.41. Regarding educational backgrounds, 139 participants (46.3%) held an associate degree, 71 (23.7%) possessed a bachelor’s degree, 60 (20%) had a master’s degree, and 30 (10%) were actively pursuing a Ph.D. All 300 university students completed the questionnaires.

For the confirmatory factor analysis, 388 university students were involved, including 230 males (59.3%) and 158 females (40.7%). Their ages ranged from 21 to 45 years, with a mean age of 24.33 and a standard deviation of 5.72. According to Soper’s [24] model, considering a structural equation model with an expected spread of results of 0.1, a desired statistical power of 0.8, 12 latent variables, 60 observed variables, and a margin of error of 0.05, the required sample size fell within the range of 200 to 947. Consequently, the questionnaire link was distributed to the students, and 300 responses were collected.

### Ethical consideration

In terms of ethical considerations, this study received approval from Alzahra University of Medical Sciences (IR.ALZAHRA.REC.1401.121), and the respondents’ names on the information form were kept confidential.

### Study instrument and variables assessment

#### Multidimensional psychological flexibility inventory (MPFI)

This instrument consists of five items (i.e., statements) for each of the six dimensions of PF and the six dimensions of PI, respectively, totaling 60 items. The dimensions include (PF/PI): Acceptance/Experiential Avoidance; Contact with the Present Moment/Lack of Contact with the Present Moment; Self as Context/Content; Defusion/Fusion; Committed Action/Inaction; and Values/Lack of Contact with Values [4]. Participants were required to rate the frequency of experiencing each statement over the past two weeks, using a 6-point scale ranging from ‘never true’ (1) to ‘always true’ (6). The English MPFI has demonstrated strong psychometric properties, including robust support for a model with 12-factor loadings onto two higher-order factors, significant correlations with the three most widely-used measures of inflexibility (i.e., AAQ, AAQ-II, and AFQ-Y), and estimated Cronbach’s α values for the different subscales ranging from 0.87 to 0.95. In the first sample, the Cronbach’s α values for the six subscales of PF range from 0.818 to 0.935, while for the six subscales of PI, the values range from 0.83 to 0.941. In the second sample, the Cronbach’s α values for the six subscales of PF range from 0.73 to 0.90, and the six subscales of PI, the values range from 0.70 to 0.89.

#### Depression, anxiety, and stress scales (DASS-21)

The DASS21 comprises 21 items, each rated on a 4-point scale, with 0 indicating ‘not at all applicable to me,’ 1 meaning ‘to some extent applicable,’ sometimes 2 referring to ‘applied to me to a significant degree or a good portion of the time,’ and 3 indicating ‘applied to me very much or most of the time.’ The Cronbach’s alpha values for these factors were reported as 0.97, 0.92, and 0.95, respectively. The results revealed that 68% of the total variance was accounted for by these three factors. In this study, the total scores for stress, depression, and anxiety factors were 9.07, 2.89, and 1.23, respectively. The Cronbach’s alpha values for these three factors were reported as 0.97, 0.92, and 0.95, respectively [25]. Samani and Jokar [26] analyzed this questionnaire, reporting test-retest validity values of 0.80, 0.76, and 0.77, and Cronbach’s alpha values of 0.81, 0.74, and 0.78 for depression, anxiety, and stress, respectively. In the first sample of the present study, Cronbach’s alpha values for depression, anxiety, and stress are 0.951, 0.84, and 0.816, respectively. In the subsequent sample of the current study, Cronbach’s alpha values for depression, anxiety, and stress are 0.87, 0.76, and 0.846, respectively.

#### AAQ-II

The AAQ-II [12], a 10-item adaptation of the original questionnaire (AAQ-I) developed by Hayes [23], was formulated to assess a construct encompassing diversity, acceptance, experiential avoidance, and psychological flexibility (PF). A higher score on the AAQ-II reflects greater psychological flexibility. Response options for the AAQ-II typically employ a Likert scale, ranging from 1 to 7, where respondents indicate their agreement with each item. The psychometric properties of the original version include results from 2816 participants across six samples, demonstrating satisfactory reliability, validity, and structural validity. The Cronbach’s alpha was 0.84 (ranging from 0.78 to 0.88), with retest reliability at 3- and 12-month intervals reported as 0.81 and 0.79, respectively. In a prior study, the AAQ-OC exhibited satisfactory internal consistency and test-retest reliability among Iranian university students [27]. In the first sample of the current study, Cronbach’s alpha for the scale was 0.839, and in the second sample, it was 0.89.

### Procedure

All participants who volunteered for this study did not receive compensation, making it a priority to obtain informed consent. Upon signing the informed consent form, participants were asked to complete an online questionnaire designed in Google Forms. The survey collected demographic characteristics such as age, gender, and educational level. Data collection commenced in March 2021 and concluded in December 2021. On average, each participant spent 30 min completing the online questionnaires. All study phases adhered to the instructions and regulations of the test.

Simultaneously with the MPFI scale, other scales that had previously been translated into Farsi and had their validity and reliability confirmed in previous studies were administered. A reverse translation technique was employed to translate Rolffs et al.’s [4] MPFI from English to Farsi. Initially, two English- Farsi translators, both native Farsi speakers, translated the MPFI scale. After a joint meeting, the two translations were reviewed, and any inconsistencies were resolved. Subsequently, the revised translation was reviewed by an expert in Farsi literature. The edited translation was then given to two translators fluent in English to ensure translation accuracy.

In the next step, an English language expert (unaware of the English version of the MPFI scale) translated the questionnaire text back into English, serving as a back-translation. Finally, in the last stage, the original version, along with the translated and re-translated versions, was presented to a third translator proficient in both English and Farsi. At this stage, the Farsi version of the MPFI was finalized and approved. After confirming the final version and resolving any ambiguities, a pilot study was conducted with 30 students to address minor issues related to the clarity of the questionnaire’s content.

### Data analysis

A comprehensive examination of the psychometric properties of the MPFI involved multiple stages. Firstly, ten experts conducted a thorough evaluation of content validity, focusing on aspects such as grammar, language use, phrase placement, and scoring. Their recommendations included calculating the Content Validity Index (CVI) for each item, determined by dividing the number of judges and assigning a rating of 3 or 4 on the Likert scale by the total number of judges. Additionally, the CVI for the relevance criterion and the global dimension were computed based on judges’ endorsements and relevant decisions, respectively. The general criterion for CVI values exceeding 0.70 was established [28].

Following this, exploratory factor analysis (EFA) was conducted on 300 cases using SPSS (Version 26). The EFA employed unweighted least squares with direct Promax rotation, and Kaiser–Meyer–Olkin (KMO) statistics were assessed to ensure factorial solution acceptability (KMO > 0.50) [29]. Bartlett’s test of sphericity was also performed, with the retention of factors and items guided by eigenvalues > 1 and factor loadings < 0.5, as indicated by the screen plot [30]. The assessment of internal consistency utilized both Cronbach’s alpha and McDonald’s omega coefficient. Concurrent validity was established by examining associations with depression, anxiety, stress, and the AAQ-II. This evaluation is pivotal as it gauges the alignment of a new measurement with an established benchmark at the same point in time. Emphasizing the significance of concurrent validity serves to underscore the instrument’s credibility and relevance in the broader context of measurement validation [31].

Simultaneously, confirmatory factor analysis (CFA) was executed on 388 cases using AMOS 23.0 to assess whether the Farsi MPFI-F exhibited a two-factor structure similar to the English version proposed by Rolffs et al. [4]. Evaluation criteria for factor loadings were in accordance with Kline’s recommendations, disregarding negative values and those below 0.40 or above 1.0. Convergent validity was assessed through the calculation of the average variance extracted (AVE), with values exceeding 0.4 considered satisfactory. The evaluation of internal consistency encompassed both a construct reliability (CR) test and a reliability test using Cronbach’s alpha, where CR values above 0.7 were considered acceptable [29]. To calculate the CR, sum the squared standardized factor loadings and divide it by the sum of squared factor loadings plus the sum of residual variances.

To calculate the AVE, aggregate the squared standardized factor loadings, and then divide the sum by the number of indicators. As for the CR, it serves as a gauge of internal consistency in scale items, akin to Cronbach’s alpha [32].

## Results

### Content validity

In the context of the Farsi MPFI, which is presumed to be a Persian-language version of a measurement tool, the Content Validity Index (CVI) is examined. The CVI is presented at two distinct levels: the item level and the scale level. Individual item CVI values range from 0.79 to 1.00. A CVI of 1.00 signifies impeccable content validity for a specific item, implying unanimous agreement among experts or evaluators that the item is highly pertinent and representative of the construct. The scale-level CVI offers a comprehensive evaluation of the content validity for the entire measurement instrument, in this case, the Farsi MPFI. The reported scale-level CVI stands at 0.86, indicating the average content validity across all items in the scale. A scale-level CVI of 1.00 would suggest flawless content validity for the entire instrument.

### Structural validity

#### Exploratory factor analysis

Data were submitted for verification of suitability to factorial analysis through the Kaiser-Meyer-Olkin (KMO) measure and Bartlett’s Test of Sphericity (BTS). The KMO value, ranging from 0 to 1, is deemed normal between 0.5 and 0.7, good between 0.7 and 0.8, very good between 0.8 and 0.9, and excellent above 0.9. The KMO value was found to be 0.915, and this value showed that the scale was suitable for factor analysis. Likewise, Bartlett’s sphericity test results (χ^2^ = 20109.442; *P* < 0.001) show that the data are interrelated and suitable for factor analysis (Table 1).

**Table 1:** Kaiser–Meyer–Olkin and Bartlett’s tests

MPFI	KMO value	χ^2^ of Bartlett’s test	P	Variance
Flexibility	0.932	8464.633	< 0.001	71.30
Inflexibility	0.929	9746.74	< 0.001	74.34
Total	0.915	20109.442	< 0.001	74.42

The eigenvalues for the identified factors in this solution are as follows (Table 2): the first-factor eigenvalue is 29.682, the second-factor eigenvalue is 11.81, the third-factor eigenvalue is 4.852, the fourth-factor eigenvalue is 4.628, the fifth-factor eigenvalue is 2.744, the sixth-factor eigenvalue is 2.33, the seventh-factor eigenvalue is 1.96, the eighth-factor eigenvalue is 1.77, the ninth-factor eigenvalue is 1.649, the tenth-factor eigenvalue is 1.58, the eleventh-factor eigenvalue is 1.38, and the twelfth-factor eigenvalue is 1.23. All factor eigenvalues exceed the threshold of 1.0, aligning with Kaiser’s rule of thumb, which recommends retaining factors with eigenvalues above this criterion [33, 34].

**Table 2:** Item-level descriptive statistics and factor loadings for MPFI items in a one-factor solution within the EFA sample

PF							PI						
Item	M	SD	r	FL	Skew	Kurtosis	Item	M	SD	r	FL	Skew	Kurtosis
1	2.88	1.39	0.30	0.61	0.11	-0.96	1	3.89	1.34	0.43	0.63	-0.77	0.91
2	3.04	1.48	0.31	0.63	-0.66	-0.48	2	3.72	1.36	0.40	0.73	-0.83	0.68
3	3.07	1.41	0.33	0.68	0.73	-0.19	3	3.84	1.29	0.44	0.73	-1.13	0.46
4	3.30	1.42	0.33	0.60	-0.43	-0.58	4	3.82	1.33	0.42	0.71	-0.86	0.89
5	3.58	1.36	0.34	0.67	-0.84	-0.32	5	3.51	1.36	0.39	0.71	-1.01	0.45
6	4.32	1.19	0.31	0.59	-0.97	0.48	6	2.94	1.38	0.30	0.73	-0.74	1.14
7	3.81	1/31	0.36	0.64	-0.59	-0.31	7	2.49	1.23	0.30	0.74	-0.26	0.10
8	4.14	1.28	0.35	0.72	-0.62	-0.44	8	2.87	1.24	0.34	0.77	-0.68	1.60
9	4.08	1.22	0.47	0.76	-0.41	-0.80	9	3.19	1.27	0.37	0.74	-0.31	1.32
10	4.29	1.23	0.42	0.53	-0.70	-1.07	10	3.06	1.24	0.43	0.74	-0.57	0.73
11	4.15	1.28	0.34	0.72	0.53	-0.68	11	3.26	1.32	0.47	0.58	-0.54	0.34
12	4.19	1.24	0.39	0.58	0.09	0.67	12	2.98	1.40	0.36	0.64	-0.15	0.16
13	4.22	1.27	0.41	0.72	0.18	0.62	13	3.12	1.37	0.38	0.59	-0.51	0.13
14	3.76	1.34	0.38	0.72	0.75	2.33	14	3.17	1.36	0.39	0.70	-0.47	-0.16
15	4.06	1.27	0.42	0.76	0.49	-0.77	15	3.01	1.41	0.37	0.64	-0.75	-0.13
16	3.30	1.42	0.35	0.53	0.61	-0.40	16	3.13	1.46	0.40	0.72	-0.39	-0.48
17	3.29	1.38	0.31	0.72	0.41	1.06	17	3.04	1.40	0.39	0.80	-0.95	-0.23
18	3.36	1.37	0.38	0.55	0.24	-1.31	18	2.99	1.45	0.35	0.76	-0.92	-0.47
19	3.35	1.29	0.37	0.72	0.65	-0.59	19	2.97	1.36	0.34	0.69	-0.82	-0.35
20	3.54	1.33	0.37	0.58	0.84	-0.52	20	3.35	1.42	0.30	0.72	-0.67	-0.49
21	3.97	1.30	0.36	0.72	0.70	-0.69	21	2.66	1.27	0.30	0.61	-0.82	-0.55
22	4.27	1.30	0.31	0.55	0.67	-0.33	22	2.82	1.36	0.38	0.68	-0.67	-0.44
23	4.34	1.22	0.35	0.60	0.45	-0.88	23	2.47	1.57	0.36	0.62	-0.23	-0.48
24	4.32	1.25	0.34	0.73	0.89	-0.84	24	2.52	1.27	0.31	0.61	0.02	-0.89
25	4.27	1.23	0.31	0.66	0.45	-0.21	25	2.51	1.28	0.32	0.76	-0.09	-0.36
26	4.35	1.32	0.30	0.57	1.14	-0.85	26	2.66	1.43	0.33	0.73	-0.22	-0.03
27	4.23	1.30	0.32	0.63	0.24	0.08	27	2.69	1.48	0.32	0.75	-0.47	0.05
28	4.26	1.30	0.32	0.60	0.65	-1.28	28	2.73	1.43	0.33	0.63	-0.41	0.01
29	4.01	1.39	0.35	0.72	0.84	1.48	29	2.73	1.38	0.30	0.71	-0.26	0.27
30	4.18	1.39	0.34	0.66	0.70	0.73	30	2.76	1.38	0.31	0.56	-0.41	-0.01

Note. Psychological flexibility = PF, Psychological inflexibility = PI, Mean = M, Standard deviation = SD, Item correlation = r, Factor loadings = FL, and Cronbach’s alpha coefficient were employed to test the internal consistency of individual MPFI scales and the total MPFI scores

### Concurrent validity

To evaluate the concurrent validity of the MPFI, Rolffs et al. [4] calculated bivariate correlations between MPFI-F and two other instruments that measure closely related psychological constructs. The set of instruments encompassed the Depression, Anxiety, and Stress Scales [25] and the AAQ-II. Table 3 displays the outcomes of concurrent validity assessment for each factor of the flexibility/inflexibility questionnaire concerning anxiety, depression, stress, and AAQ-II.

**Table 3:** Bivariate correlation coefficients of MPFI and DASS as well as MPFI and AAQ-II in the EFA sample (*n* = 300)

Variable	M	SD	α	ω	Anxiety	Depression	Stress	AAQ-II
AC	15.88	5.39	0.82	0.791	− 0.23^**^	− 0.21^**^	-0.22^**^	0.24^**^
CPM	20.65	5.07	0.87	0.872	− 0.24^**^	− 0.25^**^	-0.25^**^	0.43^**^
SCX	20.40	5.16	0.86	0.909	-0.34^**^	-0.42^**^	-0.39^**^	0.49 ^**^
CD	16.84	5.79	0.90	0.936	-0.29^**^	-0.37^**^	-0.41^**^	0.45^**^
VL	21.18	5.28	0.89	0.796	-0.35 ^**^	-0.41^**^	-0.39^**^	0.41^**^
CA	21.05	5.99	0.94	0.958	-0.37^**^	-0.47 ^**^	-0.48^**^	0.53^**^
PF	116.01	24.19	0.94	0.885	-0.33^**^	-0.41^**^	-0.42^**^	0.52^**^
EA	18.79	5.62	0.89	0.793	0.25^*^	0.21^**^	0.29^**^	-0.21^**^
LCPM	14.57	4.99	0.84	0.887	0.22^**^	0.25^**^	0.29^**^	-0.22^**^
SCT	15.56	5.67	0.88	0.826	0.50^**^	0.49^**^	0.53^**^	-0.46^**^
FU	15.49	6.04	0.90	0.895	0.50^**^	0.58^**^	0.65^**^	-0.56 ^**^
LCV	13.58	6.40	0.91	0.853	0.57^**^	0.63^**^	0.68^**^	-0.61^**^
IN	12.98	5.55	0.94	0.940	0.52^**^	0.56^**^	0.63^**^	-0.58^**^
PI	90.99	24.76	0.94	0.911	0.53^**^	0.56^**^	0.64^**^	-0.71^**^

Note. Acceptance = AC, Contact present moment = PM, Self as context = SCX, Cognitive defusion = CD, Values = VL, Committed action = CA, Psychological flexibility = PF, Experiential avoidance = EA, Lack of Contact with the Present Moment = LCPM, Self as content = SCT, Fusion = FU, Lack of contact with values = LCV, Inaction = IN, Psychological inflexibility = PI, Mean = Mean, Standard deviation = SD, Cronbach Alpha = α, McDonald’s Omega = ω, ^*^p < 0.05, **p < 0.01

As indicated in Table 3, a significant inverse association was evident between the subscales of PF and depression, anxiety, and stress (*P* ≤ 0.001). There was also a significant and positive association between the subscales of PF and AAQ-II (*P* ≤ 0.001). Moreover, a significant positive relationship was observed between the subscales of PI and measures of depression, anxiety, and stress (*P* ≤ 0.001). Furthermore, a pronounced negative correlation was identified between the subscales of PI and AAQ-II (*P* ≤ 0.001).

### Confirmatory factor analysis

The Confirmatory Factor Analysis (CFA) was employed to assess the model identified in the Exploratory Factor Analysis (EFA) and determine its fit using Goodness of Fit Indices (GFI). Two CFA models were tested using maximum likelihood estimation. The initial model examined was a one-factor model, predicting that all items would load onto a single factor representing a general MPFI factor. The analysis indicated that the single CFA factor provided a good fit for the data, with χ^2^ = 872.761; *df* = 2.238; *p* = 0.001; RMSEA = 0.06; SRMR = 0.044; TLI = 0.91; CFI = 0.90. Additionally, the AVE for each construct exceeded 0.50, and CR values substantially exceeded 0.70, confirming the convergent validity of the MPFI (Table 4).

**Table 4:** Confirmatory factor analysis for one factor solution: indices of validity and reliability

PF	items	FL	C.R	AVE	PI	items	FL	C.R	AVE
AC	1	0.84	7.59	0.58	EA	1	0.81	12.69	0.63
	2	0.74	10.63			2	0.84	13.00	
	3	0.81	11.30			3	0.85	13.13	
	4	0.75	10.91			4	0.79	12.37	
	5	0.67	-			5	0.68	-	
CPM	6	0.69	12.81	0.57	LCPM	6	0.64	10.87	0.51
	7	0.73	13.68			7	0.70	11.93	
	8	0.74	13.63			8	0.79	13.52	
	9	0.78	15.14			9	0.66	11.25	
	10	0.83	-			10	0.79	-	
SCX	11	0.69	12.28	0.57	SCT	11	0.70	13.23	0.55
	12	0.85	15.48			12	0.76	14.87	
	13	0.84	15.31			13	0.77	15.24	
	14	0.60	10.76			14	0.61	15.63	
	15	0.76	-			15	0.84	-	
CD	16	0.86	16.30	0.65	FU	16	0.86	-	0.67
	17	0.85	15.93			17	0.92	22.63	
	18	0.79	14.50			18	0.85	19.18	
	19	0.75	13.55			19	0.68	13.80	
	20	0.78	-			20	0.75	15.53	
VL	21	0.69	-	0.63	LCV	21	0.76	-	0.68
	22	0.83	13.09			22	0.82	14.96	
	23	0.87	13.51			23	0.86	16.04	
	24	0.78	12.38			24	0.88	16.06	
	25	0.79	12.46			25	0.81	14.92	
CA	26	0.79	-	0.74	IN	26	0.89	-	0.76
	27	0.85	16.96			27	0.89	23.27	
	28	0.88	17.64			28	0.88	22.65	
	29	0.90	18.18			29	0.85	20.85	
	30	0.89	17.88			30	0.85	21.03	
PF				0.61	PI				0.59

Note. Note. Acceptance = AC, Contact with the present moment = CPM, Self as context = SCX, Cognitive defusion = CD, Values = VL, Committed action = CA, Psychological flexibility = PF, Experiential avoidance = EA, Lack of Contact with the Present Moment = LCPM, Self as content = SCT, Fusion = FU, Lack of contact with values = LCV, Inaction = IN, Psychological inflexibility = PI, Factor loading = FL, ^**^P < 0.01 Note: Given that the non-standardized factor loading of each factor’s marker is set at 1, their standard error and t scores were not calculated

Subsequently, the second model explored was the second-factor model, allowing factors to intercorrelate. Although this model demonstrated a better fit than the one-factor model, with χ^2^ = 1001.512; *df* = 2.141; *p* = 0.001; RMSEA = 0.054; SRMR = 0.041; TLI = 0.92; CFI = 0.92, the first-order CFA findings were still robust.

**Fig. 2:**
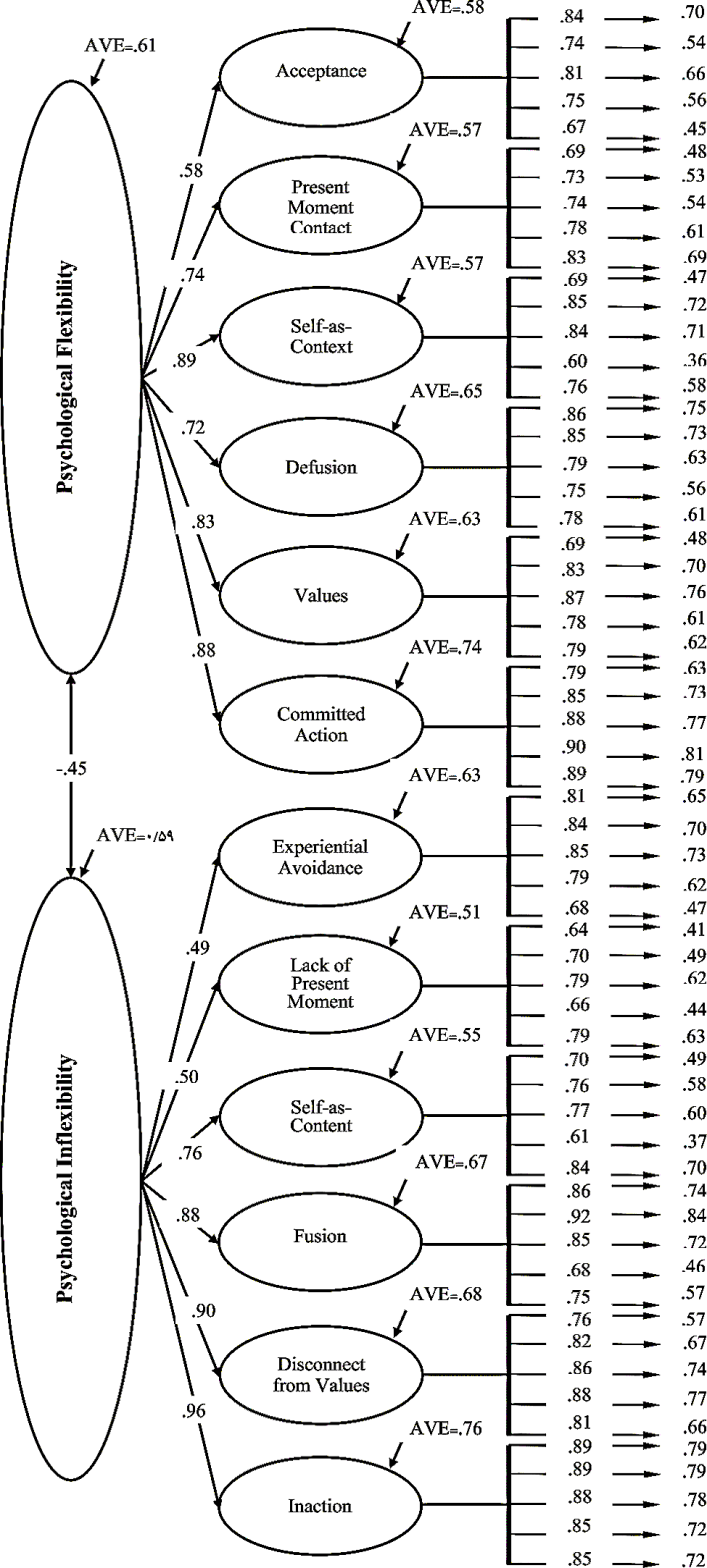
Second-order confirmatory factor analysis of MPFI

Figure 2 illustrates the results obtained from the second-order CFA, detailing factor loadings for each respective item. The utilization of both first-order and second-order CFA contributes to a comprehensive evaluation of the MPFI model’s goodness of fit, providing a nuanced understanding of the relationships among the identified factors.

## Discussion

This study makes a substantial contribution to our understanding of the psychometric characteristics of the MPFI within the Hexaflex model, with a specific focus on Iranian university students. The dual impact of our research on both theoretical and practical aspects in the realm of psychological assessment and intervention underscores its significance. Our primary contribution lies in the comprehensive examination of the Farsi version of the MPFI, affirming its robustness, reliability, and effectiveness within the unique cultural and educational context of Iranian university students. This validation is pivotal as it firmly supports the MPFI’s utilization as a valuable assessment tool for evaluating ACT within university settings.

The methodological strength of our study, incorporating both EFA and first-order and second-order CFA, adds rigor to the evaluation process. EFA enables us to investigate into the underlying structure of the MPFI in our specific population, providing a nuanced understanding of its dimensions. The subsequent application of first-order and second-order CFA models fortifies the study, offering a comprehensive assessment of the instrument’s psychometric properties. Our specific focus on Iranian university students addresses a remarkable gap in the literature, given the documented increase in depression, anxiety, and stress within this demographic. Tailored research was deemed necessary to comprehend the unique psychological processes these students experience, thereby contributing not only to academic understanding but also furnishing practical insights for mental health professionals working with university students.

In essence, our research serves as a foundational step in establishing the MPFI as a valid and reliable tool for assessing psychological flexibility within the Hexaflex model in Iranian university settings. The implications of our findings transcend this specific cultural context, providing a robust framework for future studies in diverse educational and cultural settings. This study thus contributes invaluable knowledge to the field, enhancing the potential for effective psychological assessments and interventions tailored to the unique needs of university students, particularly in regions with documented mental health challenges. Investigating our research objectives, we aimed to adapt and authenticate the Farsi rendition of the MPFI, assess its psychometric attributes, and scrutinize its factor architecture utilizing a cohort of Iranian university students. The findings from the EFA identified twelve factors, aligning with previous studies validating the MPFI in different cultural and linguistic contexts, such as Rolffs et al.‘s [4] study, the Italian version by Landi et al. [19], the French version’s validation study by Grégoire et al. [18], and the Swedish version by Tabrizi et al. [7].

Based on the Hexaflex model, these 12 factors demonstrated significant interrelationships. The six flexibility factors—acceptance, present-moment awareness, self as context, cognitive defusion, values, and engaged action—showed correlations with the six PF factors, namely experiential avoidance, absence of present-moment awareness, self as content, fusion, detachment from values, and inaction. However, despite logical correlations between the dimensions of flexibility and inflexibility, they retained distinct variances. This suggests that each flexibility dimension and its variations can offer specific insights into the impacts of psychotherapy within a cohesive model with autonomous dimensions. For instance, an individual might demonstrate enhanced acceptance without a parallel rise in committed action. While these factors are interrelated, progress in one rigidity aspect, like a diminished lack of present-moment awareness, might not necessarily coincide with advancements in others, such as defusion. The findings indicate that certain dimensions of inflexibility, such as experiential avoidance, can show improvement without a marked enhancement in the corresponding flexibility dimension, such as acceptance.

This nuanced understanding was elucidated by the EFA and correlation coefficient outcomes, pointing to two unique processes within the MPFI—flexibility on one spectrum and inflexibility on another. These observations resonate with the notions of positive and negative impacts, underscoring that flexibility and inflexibility are separate processes, rather than an excessive focus on a singular aspect. This nuanced understanding of the interplay between flexibility and inflexibility enriches the existing literature on psychological flexibility, providing a more intricate model for consideration [35]. Furthermore, this study demonstrated commendable internal consistency for the Farsi adaptation of the MPFI, consistent with previous scholarly investigations [4, 7, 17–19, 36]. The factors of acceptance, present-moment awareness, self as context, cognitive defusion, values, committed action, total PF, experiential avoidance, absence of present-moment awareness, self as content, fusion, detachment from values, inaction, and total PI all yielded Cronbach’s alpha values affirming the reliability of the instrument.

The questionnaire exhibited an acceptable level of convergent validity, as evidenced by the indices associated with each dimension of PF and PI, all surpassing the threshold of 0.50. This stands in contrast to the study by Tabrizi et al. [7], who reported convergent validity below 50 for each of the PF and PI subscales. In essence, our findings affirm the acceptable convergent validity of the Farsi version of the MPFI, aligning with the English form of the instrument. Regarding convergent validity, our data suggests that PF, along with its components, maintained a negative and statistically salient relationship with DASS (Depression, Anxiety, and Stress Scale) at a significance level of *P* ≤ 0.001. Additionally, PI and its constituent elements displayed a positive and statistically meaningful association with DASS (*P* ≤ 0.001). These findings indicate that elevated levels of PF correlate with diminished manifestations of depression, anxiety, and stress, as well as increased acceptance and action. Conversely, elevated levels of PI are associated with heightened symptoms of depression, anxiety, and stress, emphasizing the role of inflexibility in contributing to psychological distress.

A positive and notable correlation was observed between PF, including its attributes, and AAQ-II (Acceptance and Action Questionnaire-II) at *P* ≤ 0.001. Conversely, a negative and significant association was found between PI and its elements with AAQ-II (*P* ≤ 0.001). This reinforces the understanding that psychological flexibility, as measured by the MPFI, is associated with increased acceptance and action, while psychological inflexibility is linked to lower levels of acceptance and action. These findings align with and further contribute to the results reported by Kashdan and Rottenberg [1], Wetterneck et al. [37], Rolffs et al. [4], and Moroz and Dunkley [17]. The main characteristic of depressive disorder, as proposed by Kashdan and Rottenberg, is the lack of flexibility in various areas. Individuals with depression often describe prevalent mood fluctuations or a lack of joy in life and frequently perceive their surroundings as unchanging. The instruction to adopt flexible cognition by confronting dysfunctional thought patterns may mitigate depressive affect. Augmenting PF, in particular, assists patients in diminishing cognitive fusion and experiential avoidance, achieved by enhancing mindfulness and engagement with experiences. This approach prompts individuals to pivot their attention from solely regulating emotions to taking tangible steps, thereby resulting in a marked decrease in symptoms of anxiety and depression [2].

The results of the CFA showed that most fit indices and pathways were significant. The six PF factors and six PI factors, together referred to as MPFI, encompassing 60 items (30 for flexibility and 30 for inflexibility), were verified and included in the final version. This robust validation provides essential tools for ACT researchers to study treatment-induced alterations and correlate flexibility dimensions with markers of mental well-being—psychological, emotional, and social. In conclusion, our study makes a significant contribution to the understanding of psychological flexibility within the Hexaflex model, especially within the distinctive context of Iranian university students. The nuanced insights gained through EFA, first-order and second-order CFA, and the examination of specific cultural contexts enrich the existing literature on the MPFI. The Farsi version of the MPFI emerges as a valid and reliable tool for assessing psychological flexibility, providing a foundation for future studies in diverse educational and cultural settings.

### Limitations of the study and future directions

Future research would consider the constraints of this study. Firstly, the applicability of the findings might be restricted due to the somewhat uniform characteristics of certain groups studied, like students with a mean age of 24 years. Secondly, the research did not evaluate the test-retest reliability, an aspect that future studies could take into account. Third, the study was limited by its sole dependence on self-reported data, restricting it to a direct data collection approach. Fourth, the discriminant validity of the MPFI wasn’t examined. Moreover, we recognize the absence of a specific analysis of gender differences as a limitation in our current study. Moving forward, future studies could address this gap by conducting measurement invariance analyses to determine whether the scale performs similarly across genders. Additionally, authors may consider running independent t-tests to explore potential gender differences in scale responses. Lastly, as with any scale, ongoing evaluation and refinement of the MPFI will be imperative in subsequent studies to ensure its continued validity and reliability.

## Data Availability

The datasets used during the current study are available from the corresponding author upon reasonable request.
